# Residence-based inequalities among women and girls aged 15–49 years using reusable and single-use menstrual materials in Ghana: a cross-sectional study

**DOI:** 10.3389/frph.2026.1874349

**Published:** 2026-10-01

**Authors:** Patience Fakornam Doe, Yula Salifu, Joseph Lasong, Abraham Norman Nortey, Frank Offei Odonkor, Edward Odoom, Amidu Alhassan, Mustapha Amoadu

**Affiliations:** 1 Department of Public Health, School of Nursing and Midwifery, University of Cape Coast, Cape Coast, Ghana; 2 Department of Population, Family and Reproductive Health, University of Ghana, Legon, Ghana; 3 Department of Population and Reproductive Health, University for Development Studies, Tamale, Ghana; 4 Department of Community Medicine, University of Cape Coast, Cape Coast, Ghana; 5 Department of Adult Health, University of Cape Coast, Cape Coast, Ghana; 6 Department of Nursing, All Nations University, Koforidua, Ghana; 7 Biomedical and Clinical Research Centre, University of Cape Coast, Cape Coast, Ghana

**Keywords:** health inequalities, menstrual health, menstrual materials, reusable pad sustainability, rural -urban disparities

## Abstract

**Background:**

Menstrual health is a critical component of sexual and reproductive well-being, yet inequalities in access to appropriate menstrual materials persist, particularly in low- and middle-income countries (LMICs). In Ghana, national progress in menstrual material use has been reported, but residence-based disparities remain insufficiently examined. This study assessed trends and rural–urban inequalities in the use of reusable and single-use menstrual materials among women and girls aged 15–49 years in Ghana.

**Methods:**

A repeated cross-sectional design was employed using nationally representative data from the 2016 Performance Monitoring for Action (PMA) survey and the 2017/18 Multiple Indicator Cluster Survey (MICS). Analysis was conducted using the WHO Health Equity Assessment Toolkit (HEAT). Survey data were accessed from the WHO HEAT platform and managed in Microsoft Excel prior to upload.

**Results:**

Reusable menstrual material use remained unchanged at 12.6% between 2016 and 2018. Single-use materials predominated (85.8% in 2016; 85.3% in 2018). Rural reusable use (21.3%; 18.0%) substantially exceeded urban use (5.5%; 7.4%). The absolute rural–urban difference narrowed from −15.8% to −10.6% for reusable, and 16.7% to 11.0% for single-use materials.

**Conclusion:**

Substantial and persistent residence-based inequalities in menstrual material use exist in Ghana. The study does not advocate for reusable over single-use materials, but calls for expanding safe, informed material choice across residential settings. Equity-responsive policies addressing affordability, access, and WASH infrastructure are urgently needed.

## Introduction

Menstrual health is an essential component of sexual and reproductive well-being and a recognised public health priority. Safe and acceptable menstrual material use supports dignity, participation, and health across the life course (1). Globally, an estimated 1.9 billion women and girls menstruate (2), yet more than 500 million lack access to adequate menstrual health resources (3). Persistent socioeconomic and geographic inequalities, with disadvantaged groups relying disproportionately on reusable or improvised materials under unsafe conditions (4–6), underscore the public health importance of this issue.

In sub-Saharan Africa, menstrual material use is shaped by poverty (6, 7), infrastructure gaps (8–10), and sociocultural norms (11). Single-use products remain inaccessible for many women and girls (12–14), particularly in rural and low-income settings. Limited access to clean water (15–17), privacy (18, 19), and disposal facilities constrains safe reuse practices and increases infection risk (17). Adolescents and women of reproductive age face compounded vulnerabilities, including stigma (20), misinformation, and constrained purchasing power (11, 21–23).

In Ghana, 97% of girls used some form of absorbent material during menstruation: 58.6% commercial sanitary pads, 30.5% cloth, 3.7% cotton, 4.2% tissue paper, and 3.0% reported using none (24). An estimated 13% reported using reusable materials during their most recent menstrual period (25). Structural inequities in income, sanitation coverage, and market availability shape these patterns. Cost (26), limited availability (27), and poor market access (28) constitute major barriers. Recent evidence from the 2022 Ghana DHS using decomposition analysis confirms that household wealth and education account for the majority (63%) of rural–urban inequalities in modern menstrual product use (29).

Despite growing attention to menstrual health, evidence on progress and equity in Ghana remains fragmented. This study examines progress and inequalities in reusable and single-use menstrual material use among women and girls aged 15–49 years in Ghana using WHO HEAT and provides the sociodemographic context necessary for interpreting observed patterns.

## Methods

### Study design and data sources

This study employed a repeated cross-sectional design using nationally representative secondary data from the 2016 PMA survey and the 2017/18 MICS conducted in Ghana (30, 31). Both surveys employed stratified two-stage cluster sampling to ensure nationally and sub-nationally representative estimates.

Both surveys covered all ten administrative regions of Ghana at the time of data collection. Primary sampling units (PSUs) were enumeration areas stratified by urban/rural status and region, selected with probability proportional to size from the Ghana Statistical Service master sampling frame. Urban localities were defined as those with a resident population of ≥5,000, per Ghana Statistical Service classification. Survey data were accessed via the WHO HEAT platform (https://www.who.int/data/inequality-monitor/assessment_toolkit), with the PMA 2016 dataset additionally archived at https://www.pmadata.org, and the MICS 2017/18 dataset at https://mics.unicef.org/surveys.

### Study population and variables

The analytical sample comprised women and girls aged 15–49 years who were currently menstruating (not pregnant, not postpartum, and having menstruated in the past month) and who provided valid responses on menstrual material type. Women who were pregnant, postpartum, amenorrhoeic, or who did not report menstruation status were excluded, as were respondents with missing responses on the material use variable. The 2016 PMA yielded approximately 2,282 menstruating women; the 2017/18 MICS yielded approximately 3,017. Both surveys asked currently menstruating women to identify material used during their most recent menstrual period; responses were mapped to reusable (cloth, homemade or purpose-made pads) and single-use (commercially produced disposable pads). A small residual (1.6% in 2016) used other materials and is retained in the denominator but not classified under either primary outcome (30, 31).

### Data management and analytical procedures

Data were accessed from the WHO HEAT platform (https://www.who.int/data/inequality-monitor/assessment_toolkit), the PMA data archive (https://www.pmadata.org), and the MICS data repository (https://mics.unicef.org/surveys). Variable harmonisation and dataset preparation were conducted in Microsoft Excel (Version 2407). The menstrual material use variable was not a built-in HEAT indicator and was derived from individual survey questionnaire items, categorised into reusable and single-use, and uploaded as a custom variable to the HEAT upload module. Missing data on the menstrual material use variable were excluded from the denominator (fewer than 2% of eligible respondents in both surveys). Pregnant, postpartum, and non-menstruating women were excluded at the eligibility-filter stage prior to analysis and were not imputed. No additional re-categorisation was applied beyond standard survey-defined categories. Survey-supplied sample weights were applied to all estimates. All tabulations and cross-checks were performed in Excel prior to HEAT upload. The HEAT platform performed all inequality calculations (32, 33).

### Data analytics and inequality measures

Residence-based inequalities were assessed using four standard summary measures (32, 33). Absolute inequality was quantified using the Difference (D; urban minus rural prevalence), relative inequality using the Ratio (R; urban/rural prevalence), and population-level impact using the Population Attributable Fraction (PAF) and Population Attributable Risk (PAR). Confidence intervals (CIs) for D and R are not generated by WHO HEAT for two-subgroup residence comparisons in the custom-upload mode; this is a known platform limitation and is acknowledged in Table 1 footnotes and the Limitations section. Although WHO HEAT supports stratification by multiple equity dimensions, the primary analysis is restricted to residence, reflecting both the study's analytical focus and a constraint of the HEAT custom-upload functionality at the time of analysis.

**Table 1:** Summary measures of residence-based inequalities in menstrual material use among women and girls aged 15–49 years in Ghana, 2016–2018.

		Reusable menstrual materials						Single-use menstrual materials					
		2016			2018			2016			2018		
Dimension	Measure	Est. (%)	CI-LB	CI-UB	Est. (%)	CI-LB	CI-UB	Est. (%)	CI-LB	CI-UB	Est. (%)	CI-LB	CI-UB
Place of residence	D	−15.8	NA^a^	NA^a^	−10.6	NA^a^	NA^a^	16.7	NA^a^	NA^a^	11.0	NA^a^	NA^a^
	PAF	0.0	0.0	0.0	0.0	0.0	0.0	8.8	8.8	8.8	5.7	5.7	5.7
	PAR	0.0	0.0	0.0	0.0	0.0	0.0	7.6	7.5	7.6	4.8	4.8	4.9
	R^b^	0.3	NA^a^	NA^a^	0.4	NA^a^	NA^a^	1.2	NA^a^	NA^a^	1.1	NA^a^	NA^a^

National sample: 2016 PMA n2,282; 2017/18 MICS n3,017 currently menstruating women aged 15–49 years (unweighted). Weighted estimates used throughout.

D, absolute difference (urban−rural); R, ratio (urban/rural); PAF, population attributable fraction; PAR, population attributable risk.

a Confidence intervals for D and R are not generated by WHO HEAT for two-subgroup residence comparisons in the custom-upload mode; this is a known platform limitation.

b R is the urban-to-rural ratio as computed by HEAT. Rural-to-urban ratio was 3.9 in 2016 and 2.4 in 2018.

### Ethical considerations

Ethical approval for the 2016 PMA survey was obtained from the Ghana Health Service Ethics Review Committee and the Institutional Review Board of Johns Hopkins Bloomberg School of Public Health. Ethical approval for the 2017/18 MICS was obtained from the Ghana Health Service Ethics Review Committee. Informed consent was obtained from all participants prior to data collection. No additional ethical approval was required for this secondary analysis of anonymised publicly available data.

## Results

### National prevalence of reusable and single-use menstrual materials in Ghana

At the national level, menstrual material use remained largely unchanged between 2016 and 2018. The prevalence of reusable materials was stable at 12.6% in both years. Single-use materials dominated, declining marginally from 85.8% to 85.3% (Figure 1).

**Figure 1:**
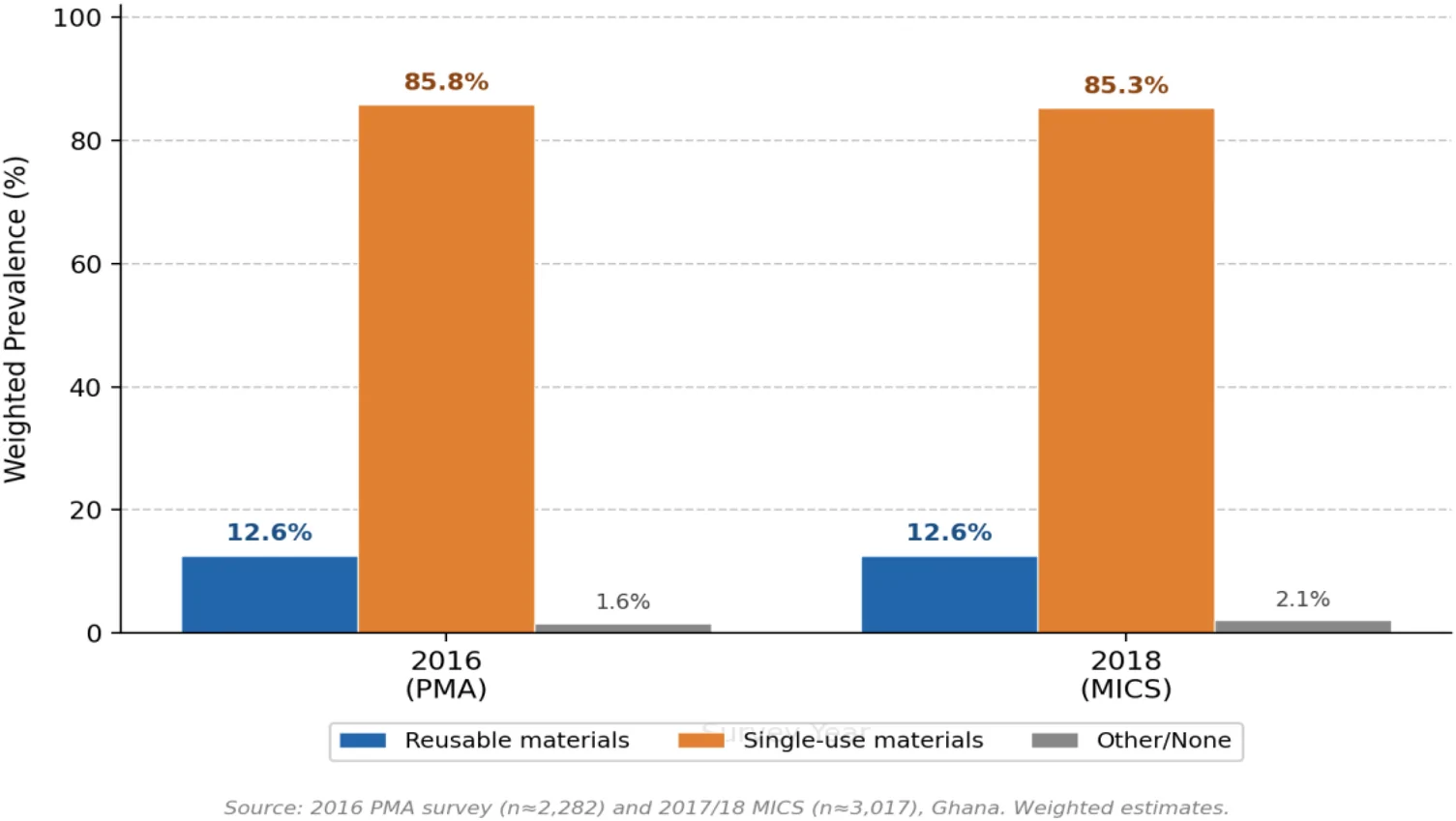
National prevalence of menstrual material use among women and girls aged 15–49 years in Ghana, 2016 and 2018.

### Rural–urban inequalities in the use of reusable menstrual materials

Reusable menstrual material use showed a clear and persistent rural–urban gradient. In 2016, rural prevalence was 21.3% versus 5.5% in urban areas; by 2018, rural use declined to 18.0% while urban use rose to 7.4%. Rural women and girls were approximately 2.4 to 3.9 times more likely to use reusable materials than their urban counterparts (Figure 2).

**Figure 2:**
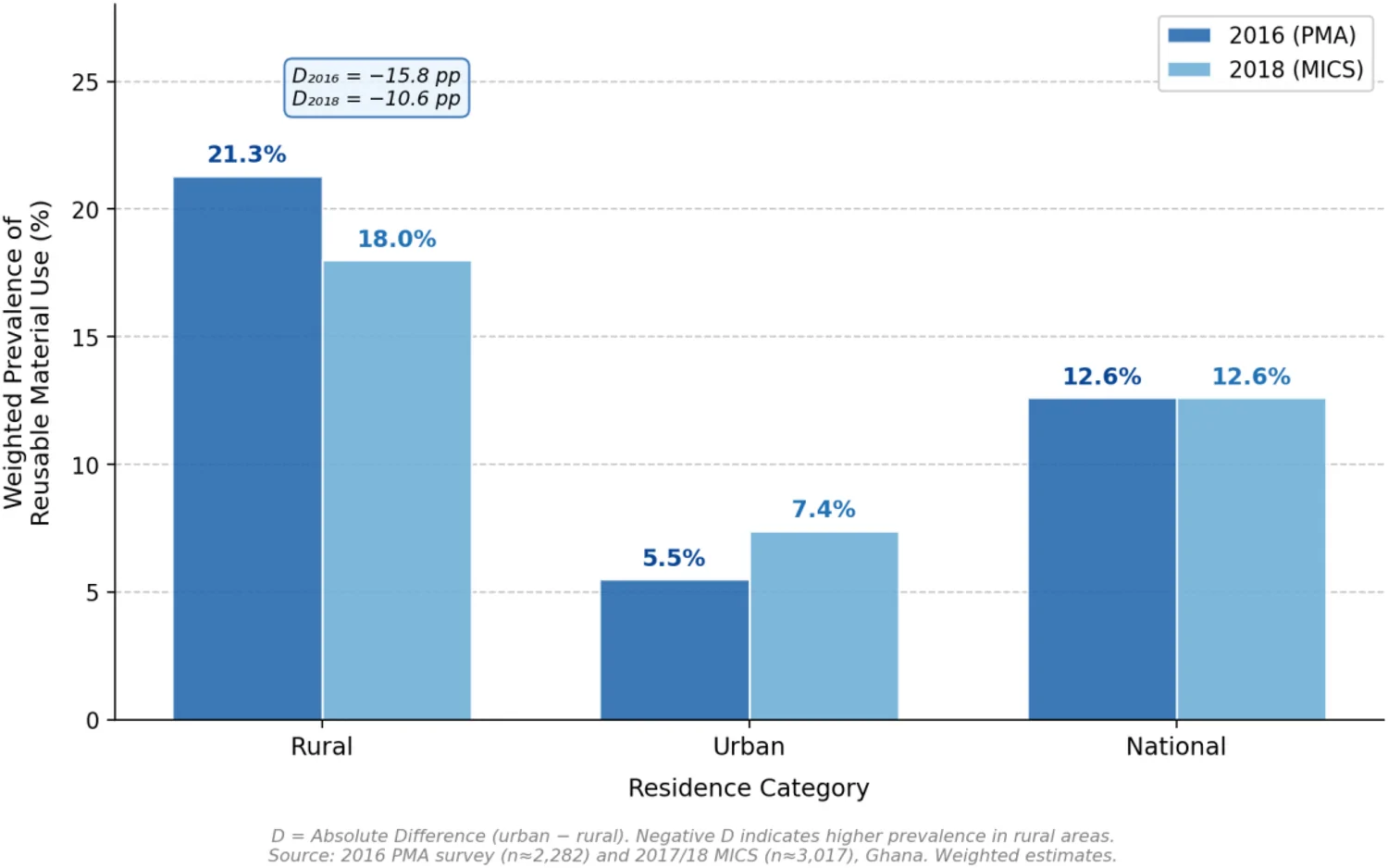
Rural–urban inequalities in reusable menstrual material use among women and girls aged 15–49 years in Ghana, 2016 and 2018.

### Rural–urban inequalities in the use of single-use menstrual materials

Single-use material use displayed the opposite distribution. Urban use was 93.3% in 2016 and 90.7% in 2018, compared with 76.6% and 79.6% in rural areas. Although the gap narrowed marginally, substantial residence-based inequality persisted (Figure 3).

**Figure 3:**
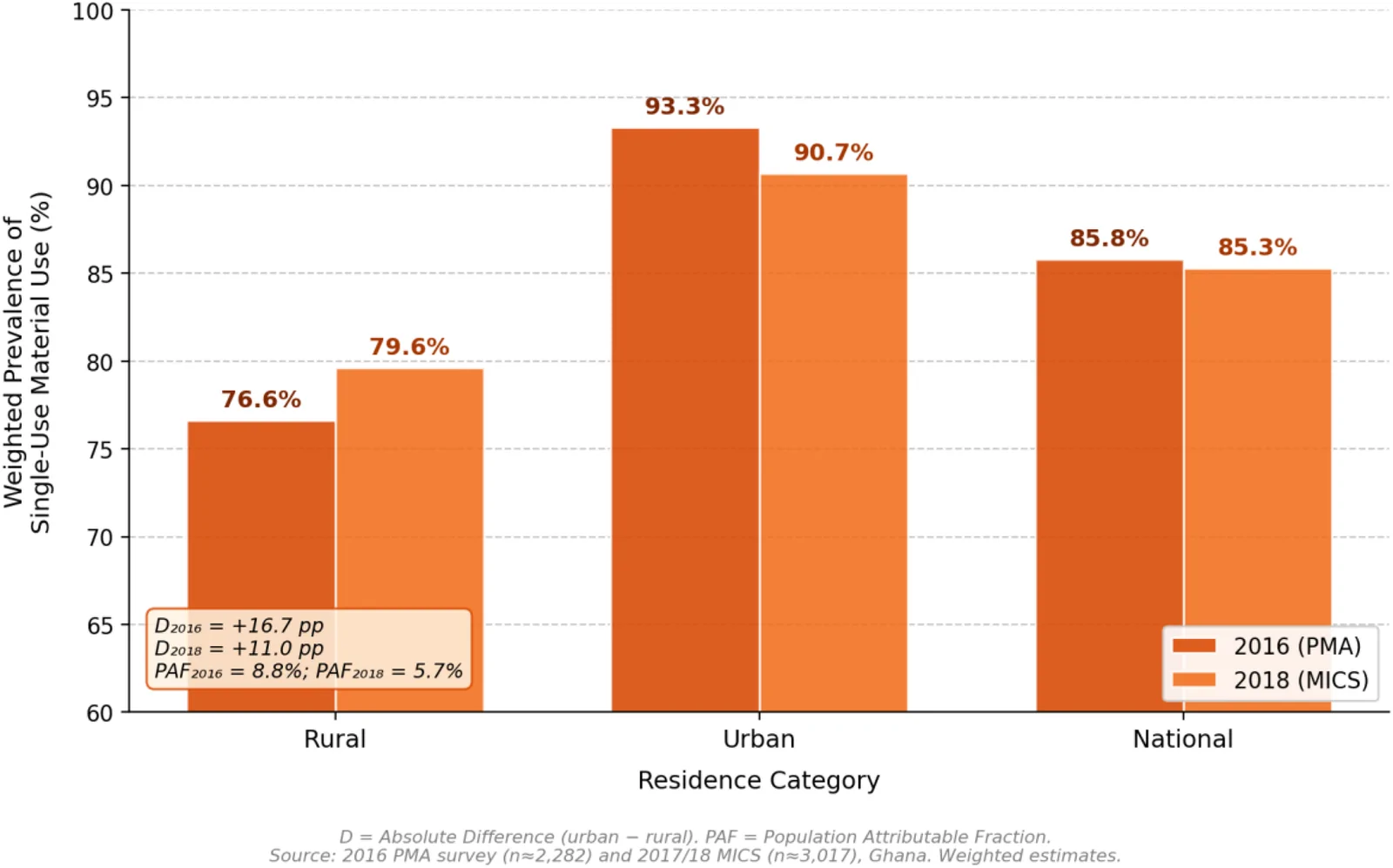
Rural–urban inequalities in single-use menstrual material use among women and girls aged 15–49 years in Ghana, 2016 and 2018.

### Summary measures of residence-based inequalities, 2016–2018

For reusable materials, D was −15.8% in 2016 and −10.6% in 2018, confirming rural concentration. The Ratio (urban/rural) was 0.3 in 2016 and 0.4 in 2018 (rural prevalence was approximately 3.9- and 2.4-times urban prevalence respectively). PAF and PAR were zero, reflecting indicator directionality configuration in HEAT. For single-use materials, D declined from 16.7% to 11.0%; PAF from 8.8% to 5.7%; PAR from 7.6% to 4.8% (Table 1).

## Discussion

The purpose of this study was to examine patterns of residence-based inequalities in the use of reusable and single-use menstrual materials among women and girls aged 15–49 years in Ghana between 2016 and 2018. Findings showed minimal national change over time, with reusable material use remaining constant at 12.6% and single-use materials continuing to dominate despite a marginal decline. Pronounced residence-based inequalities persisted, with reusable materials more commonly used in rural areas, and single-use materials consistently more prevalent in urban settings.

The observed stability in national prevalence suggests that menstrual material use in Ghana is shaped by entrenched structural and economic conditions rather than short-term change. Continued dominance of single-use materials aligns with evidence from LMICs, where disposable pads are widely perceived as safer, more hygienic, and socially acceptable, particularly in formal and urban settings (27, 34, 35). The lack of growth in reusable material use contrasts with sustainability-oriented policy narratives, suggesting awareness alone is insufficient without parallel improvements in WASH infrastructure, privacy, and cultural acceptance (17).

The pronounced rural–urban gradient is consistent with prior empirical work. Higher rural reliance on reusable materials reflects affordability constraints, limited retail availability, and weaker supply chains across sub-Saharan Africa (36, 37). Higher urban use of single-use materials mirrors evidence that urban residence is associated with improved market access, higher income, and commercial advertising exposure (6). The modest convergence between 2016 and 2018 suggests gradual diffusion of disposable products into rural areas, though progress remains uneven. Declining inequality indices for single-use materials suggest that improvements in rural uptake contributed meaningfully to national coverage gains.

This analysis makes several distinct contributions to knowledge. First, it provides the first equity-focused quantification of both reusable and single-use menstrual material use in Ghana using the standardised HEAT framework, generating internationally comparable inequality metrics that go beyond descriptive subgroup comparisons. Second, by analysing two consecutive nationally representative surveys, the study establishes a baseline trajectory for 2016–2018. Third, the analysis demonstrates that the rural–urban gradient in reusable use is substantially larger than the national average implies. Fourth, the 12.6% national prevalence is consistent with, and validates, the 13% figure reported by Anaba et al. from the 2017/18 MICS (25). Complementing these findings, recent decomposition analysis of the 2022 Ghana DHS confirms that household wealth and education account for the majority (63%) of rural–urban inequalities in modern menstrual product use in Ghana and identifies media exposure and community-level education as additional significant determinants (29).

Cross-national evidence from a 2024 analysis of 42 surveys in LMICs reported wide variation in reusable menstrual material use, ranging from less than 5% in some upper-middle-income country settings to more than 50% in some low-income sub-Saharan African countries where disposable products are prohibitively expensive (38). Ghana's 12.6% national prevalence is broadly consistent with lower-middle-income country patterns (38). It is lower than reusable use rates reported in Ethiopia (39) and Uganda (40), but higher than rates documented in Kenya and South Africa, where market penetration of disposables is more advanced (38, 41, 42). In West Africa, data suggest reusable cloth use of approximately 15%–25% in rural populations in comparable settings, placing Ghana's rural estimate (18%–21%) within a plausible regional range (38, 43). These comparisons suggest that Ghana's pattern reflects a regional distribution shaped by moderate WASH coverage, growing urban markets for disposable products, and persistent rural poverty (6, 37, 38). Notably, in countries where reusable use is widespread, high prevalence is typically attributable to the near-complete unaffordability of disposable pads rather than informed sustainable choice, underscoring the equity framing of this analysis (6, 25, 37, 38).

More recent nationally representative data from Ghana are expected to provide updated estimates on menstrual material use. These data were not yet incorporated into the WHO HEAT built-in indicator library at the time this analysis was conducted, and the custom-upload approach used here precludes direct integration of new survey rounds without re-uploading and reprocessing. Evidence from the 2022 Ghana DHS indicates that 88.0% of women aged 15–49 used modern menstrual products nationally; of modern users, 97.6% used disposable products and 2.4% used sustainable (reusable) products, indicating that while overall modern product use has grown since 2018, reusable modern product use remains very low (29). Rural use of modern products (76.0%) continues to lag substantially behind urban use (29, 44). Evidence from a 2024 cross-national analysis documents that reusable material use tends to be highest in countries with the lowest household incomes, weakest market infrastructure, and lowest female education levels (38). Settings where menstrual health programmes have been scaled with dedicated WASH support, product quality standards, and sustained supply chains, such as pilot interventions in Uganda, have demonstrated that multi-component menstrual health interventions are acceptable and feasible, and show potential to improve menstrual hygiene management and school attendance when enabling conditions are in place (45, 46). These findings carry a clear policy message for Ghana: expanding access to safe, quality-assured reusable products in rural areas is feasible, but only when accompanied by adequate WASH infrastructure, hygiene education, and product quality assurance (6, 17, 45, 46).

Defining truly sustainable reusable menstrual materials: The survey response category “reusable” encompasses a heterogeneous range of products. Three types can be distinguished. First, improvised cloth products, pieces of old fabric or rags repurposed for menstrual absorption, are the most common form in rural Ghana. These are typically made from cotton or polyester-cotton blends and pose hygiene risks if not adequately washed, dried, and stored. Second, purpose-made reusable cloth pads are machine-sewn multi-layer pads designed specifically for menstrual use, typically constructed from absorbent cotton flannel inner layers with a water-resistant outer shell (often polyurethane laminate/PUL fabric); examples include AFRIpads and locally produced NGO-distributed pads. These can last 1–3 years with proper care and cost substantially less over time than repeated disposable purchases (45). Third, menstrual cups are bell-shaped devices made from medical-grade silicone, rubber, or latex that collect rather than absorb menstrual flow; they last up to 10 years and are widely considered the most environmentally sustainable option (45). Truly sustainable reusable materials require: (i) body-safe, durable construction; (ii) access to clean water and soap for washing; (iii) ventilated drying space; (iv) clean, dry storage; and (v) timely replacement. Without these enabling conditions, even purpose-made products can pose health risks (17, 18, 47).

The association between enhanced economic status and single-use pad use warrants careful interpretation. This study does not advocate for reusable over single-use materials, nor does it recommend reusable materials as universally preferable. The critical equity concern is constrained choice: rural women disproportionately use reusable cloth not by preference, but due to the unaffordability and inaccessibility of single-use alternatives. Addressing this inequity requires expanding access and reducing financial barriers to both product types. For urban residents, strategies to encourage informed adoption of quality reusable products, where desired, could include: (i) awareness campaigns highlighting the cost-effectiveness and environmental benefits of purpose-made reusable pads and menstrual cups; (ii) inclusion of reusable products in school and workplace health programmes; (iii) public procurement for distribution to low-income urban households; and (iv) menstrual health education building skills for safe reuse (17, 18, 45, 46). The evidence base for behaviour change around reusable product adoption in urban settings is nascent and represents an important priority for future research (29). Behavioural interventions should consider the interaction between individual practices and the broader infrastructural and contextual conditions that enable safe menstrual hygiene behaviours (48, 49).

Two conceptual tensions warrant explicit clarification. First, while higher rural reusable use might appear aligned with sustainability goals, this equity analysis indicates it reflects constrained choice and limited market access rather than informed sustainable preference. Second, single-use products bring challenges including recurrent cost dependency and solid waste disposal burdens. Menstrual health interventions should be evaluated through both equity and life-cycle sustainability lenses.

## Implications of the study

The persistence of residence-based inequalities calls for coordinated action. The Ministry of Health and Ghana Health Service should mainstream menstrual health within reproductive and adolescent health policies, with explicit equity targets for rural populations. The Ministry of Education has a critical role in integrating menstrual support into school health programmes. The Ministry of Gender, Children and Social Protection can align menstrual product provision with social protection schemes. Development partner engagement (WHO, UNICEF) remains essential for technical guidance and monitoring.

Community health nurses, midwives, teachers, and district environmental health officers should be equipped to deliver comprehensive menstrual health education. District Assemblies play a pivotal role in improving WASH infrastructure. Future analyses using individual-level microdata should explore intersecting inequalities across wealth, education, age, and region simultaneously. Updated analyses using the Ghana DHS 2022 and subsequent PMA rounds are urgently needed (29, 44).

### Strengths and limitations

This study is strengthened by its use of nationally representative data from two consecutive survey rounds, its application of WHO HEAT for standardised inequality measurement, and its provision of a baseline trajectory for residence-based inequalities in menstrual material use in Ghana. Important limitations include: the age of the data (8–10 years); restriction to residence as the sole equity stratifier; unavailability of CIs for D and R in HEAT custom-upload mode; heterogeneity of the reusable category; inability to capture dual or mixed material use; absence of WASH stratification; and the inferential nature of structural explanations. The repeated cross-sectional design restricts inference to associations. Findings should be complemented by longitudinal and mixed-methods research.

## Conclusions

This study provides an important baseline characterisation of residence-based inequalities in menstrual material use in Ghana for 2016–2018. Progress was minimal and unevenly distributed. Reusable materials remained disproportionately concentrated in rural settings, reflecting affordability, market availability, and WASH infrastructure constraints. This study does not advocate for reusable over single-use materials; it advocates for expanding safe, informed material choice across residential settings. Complementary evidence from the 2022 Ghana DHS confirms that wealth and education remain major structural drivers of menstrual product inequalities (29). Updated analyses using Ghana DHS 2022 data and subsequent PMA rounds are urgently needed to track progress since 2018 (29, 44).

## Data Availability

Publicly available datasets were analyzed in this study. This data can be found here: https://www.who.int/data/inequality-monitor/assessment_toolkit.
